# Orthogonal chemical functionalization of patterned gold on silica surfaces

**DOI:** 10.3762/bjnano.6.233

**Published:** 2015-12-01

**Authors:** Francisco Palazon, Didier Léonard, Thierry Le Mogne, Francesca Zuttion, Céline Chevalier, Magali Phaner-Goutorbe, Éliane Souteyrand, Yann Chevolot, Jean-Pierre Cloarec

**Affiliations:** 1 Université de Lyon, Institut des Nanotechnologies de Lyon, site École Centrale de Lyon, CNRS UMR 5270, 36 Avenue Guy de Collongue, 69134 Écully, France; 2 Université de Lyon, Institut des Sciences Analytiques, Université Claude Bernard Lyon 1 / CNRS / ENS de Lyon, CNRS UMR 5280, 5 rue de la Doua, 69100 Villeurbanne, France; 3 Université de Lyon, École Centrale de Lyon, Laboratoire de Tribologie et Dynamique des Systèmes, CNRS UMR 5513, 36 Avenue Guy de Collongue, 69134 Écully, France; 4 Laboratoire Nanotechnologies & Nanosystèmes (UMI-LN2 3463), Université de Sherbrooke - CNRS - INSA de Lyon - ECL - UJF-CPE Lyon, Université de Sherbrooke, 3000 Boulevard de l’Université, Sherbrooke, Québec J1K 0A5, Canada,; 5 LTM/CNRS/RENATECH, 17 rue des martyrs, 38054 Grenoble, France

**Keywords:** characterization, self-assembled monolayer, surface functionalization, ToF–SIMS, XPS

## Abstract

Single-step orthogonal chemical functionalization procedures have been developed with patterned gold on silica surfaces. Different combinations of a silane and a thiol were simultaneously deposited on a gold/silica heterogeneous substrate. The orthogonality of the functionalization (i.e., selective grafting of the thiol on the gold areas and the silane on the silica) was demonstrated by X-ray photoelectron spectroscopy (XPS) as well as time-of-flight secondary ion mass spectrometry (ToF–SIMS) mapping. The orthogonal functionalization was used to immobilize proteins onto gold nanostructures on a silica substrate, as demonstrated by atomic force microscopy (AFM). These results are especially promising in the development of future biosensors where the selective anchoring of target molecules onto nanostructured transducers (e.g., nanoplasmonic biosensors) is a major challenge.

## Introduction

**Figure 1:**
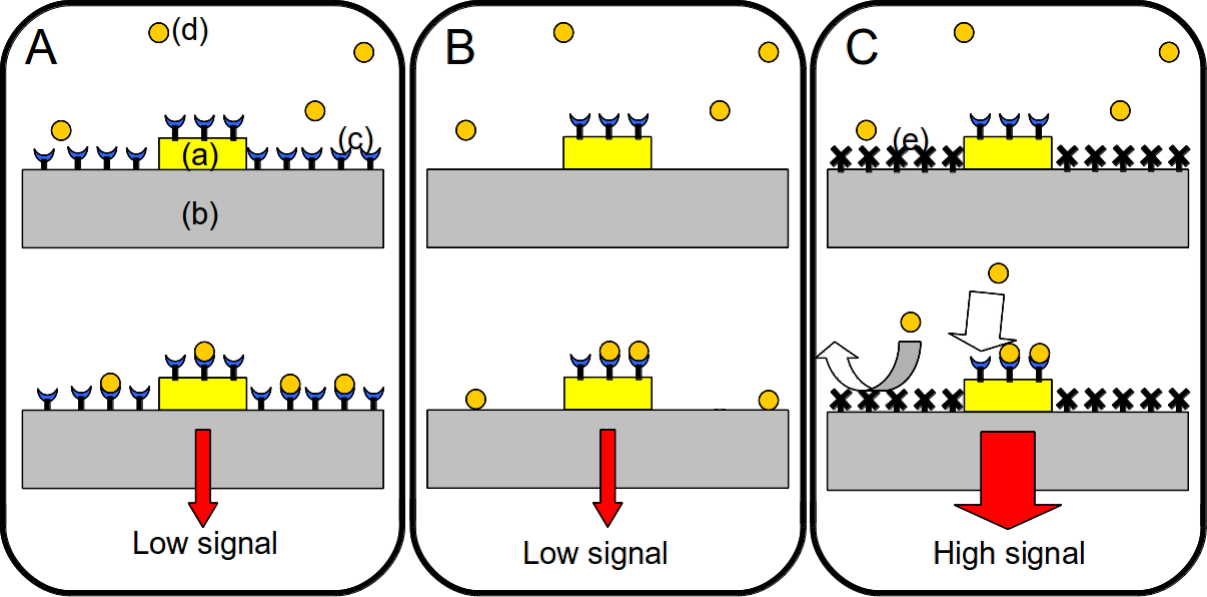
Schematic representation of the use of orthogonal functionalization techniques to enhance the sensitivity of a plasmonic biosensor (with a constant number of molecules). (A) Functionalization is uniform over the entire surface. The immobilization of probes (c) onto the entire surface, including the LSPR zone (a) and silica substrate (b). The targets (d) are captured far from the LSPR area. (B) Only the nanotransducer is functionalized. Selective immobilization of probes onto the LSPR area (a) only. The targets can absorb onto the silica substrate (b) far from the LSPR zone. (C) Orthogonal functionalization on the nanotransducer and surrounding surface. Selective immobilization of probes onto the LSPR area only, and selective nonfouling treatment (e) on the silica substrate. The targets only bind to the enhanced detection area.

The orthogonal self-assembly of different molecules onto a patterned substrate was first demonstrated in 1989 by Whitesides and co-workers [1]. Recently, especially with the development of localized surface plasmon resonance (LSPR) biosensors, this topic has become a major focus [2–8]. Indeed, LSPR transduction is expected to yield enhanced signal as compared to classical SPR transduction. However, the enhancement of the LSPR limit of detection is effective only if the molecular targets reach the surface of the metallic LSPR active zones. When dealing with a low concentration of molecular targets, it is necessary to reduce nonspecific adsorption of targets outside of these LSPR active zones, and to increase the specific capture of targets onto LSPR hot spot areas. Orthogonal surface chemical functionalization appears to enable such directed anchoring of target biomolecules (Figure 1) [6,8–9].

Despite the aforementioned publications, there is still much to be investigated regarding the orthogonal functionalization of patterned metal on dielectric surfaces for even greater enhancement of LSPR-based biosensors. First, the orthogonality of the functionalization is often assumed from “end of process measurements” (i.e., SPR signal readout occurs after target immobilization) rather than directly characterized prior to target immobilization. Second, while biotinylated poly(ethylene glycol) [5–8] may be well suited to immobilize some biomolecules (avidin derivatives), it is worth considering other surface chemistries. For instance, carboxylic acid-based [10–21], amine-based [22–26] or other [27–28] self-assembled monolayers may provide a higher diversity of potential biomolecules to immobilize. Shorter spacer chains (e.g., short alkyl chains) may also be useful to immobilize the target as close to the metal surface (i.e., the maximum intensity of the evanescent field) as possible. If the molecules used for the orthogonal functionalization are truly selective for each material, it can be expected that the functionalization of both may be performed simultaneously, thus simplifying the whole process.

Therefore, this paper presents a facile single-step orthogonal functionalization protocol to selectively bind different thiols and silanes (mixed in organic solvent at room temperature) onto the gold and silica areas of a patterned surface. The chemical functionalization was verified by direct characterization using XPS and ToF–SIMS mapping. To this end, microscale gold structures were used to evaluate the different materials separately (especially for XPS characterization, whose spatial resolution is on the order of 10 µm) and combined with perfluorinated thiols and silanes that give a strong fluorine signal both in XPS and ToF–SIMS measurements. Finally, an orthogonal functionalization with biologically pertinent molecules (antifouling poly(ethylene glycol) silane and biotinylated thiols) was used for the selective immobilization of proteins onto metallic nanostructures relevant to the development of LSPR biosensors and characterized by atomic force microscopy (AFM).

## Results and Discussion

**Figure 2:**
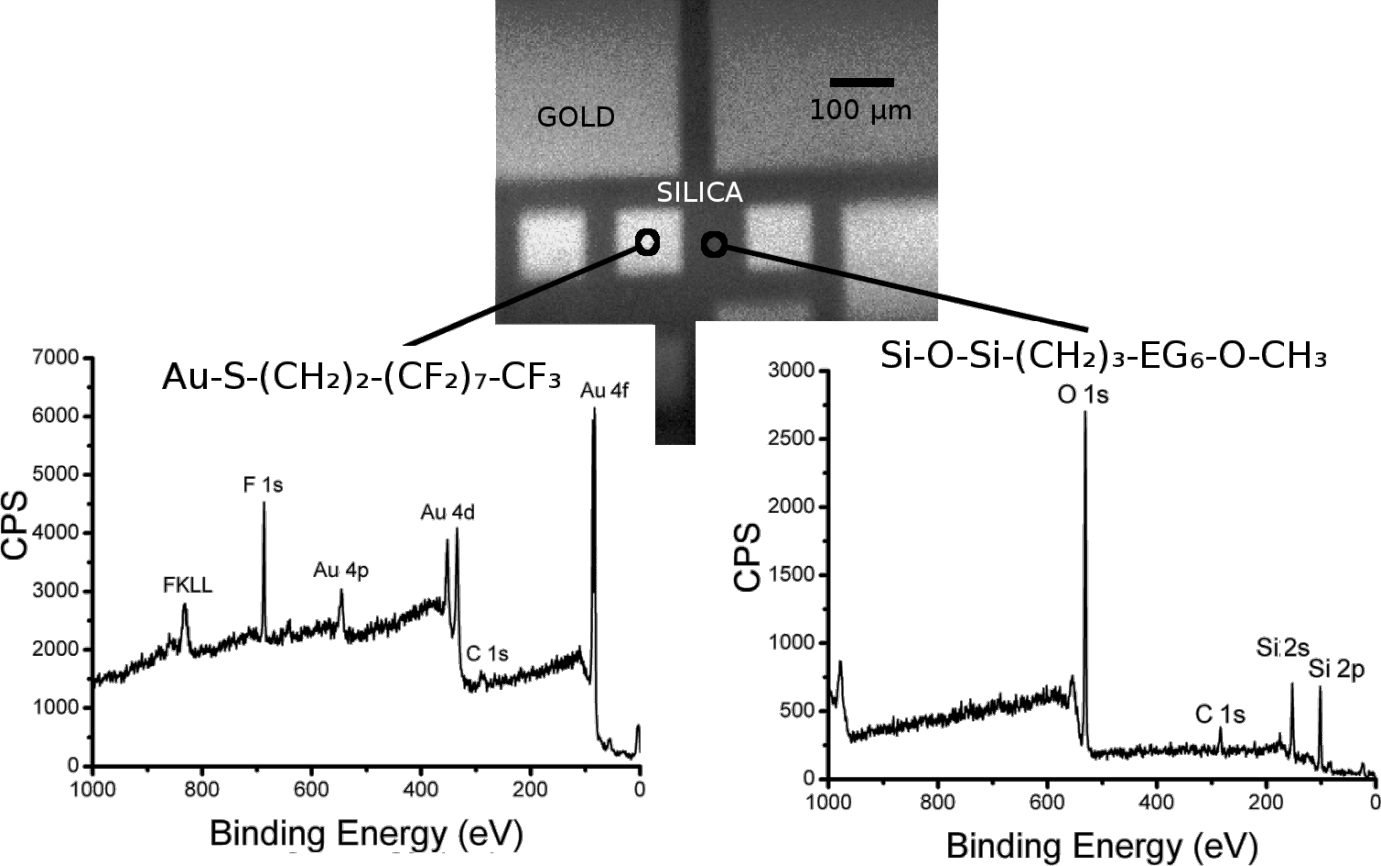
An SXI image and XPS spectra of a micropatterned gold on silica substrate sample, orthogonally functionalized with F-thiol and PEG/Si. The analyzed areas for the spectra were roughly 10 μm and their approximate localization is indicated on the image. The scale bar in the image is 100 µm.

**Figure 3:**
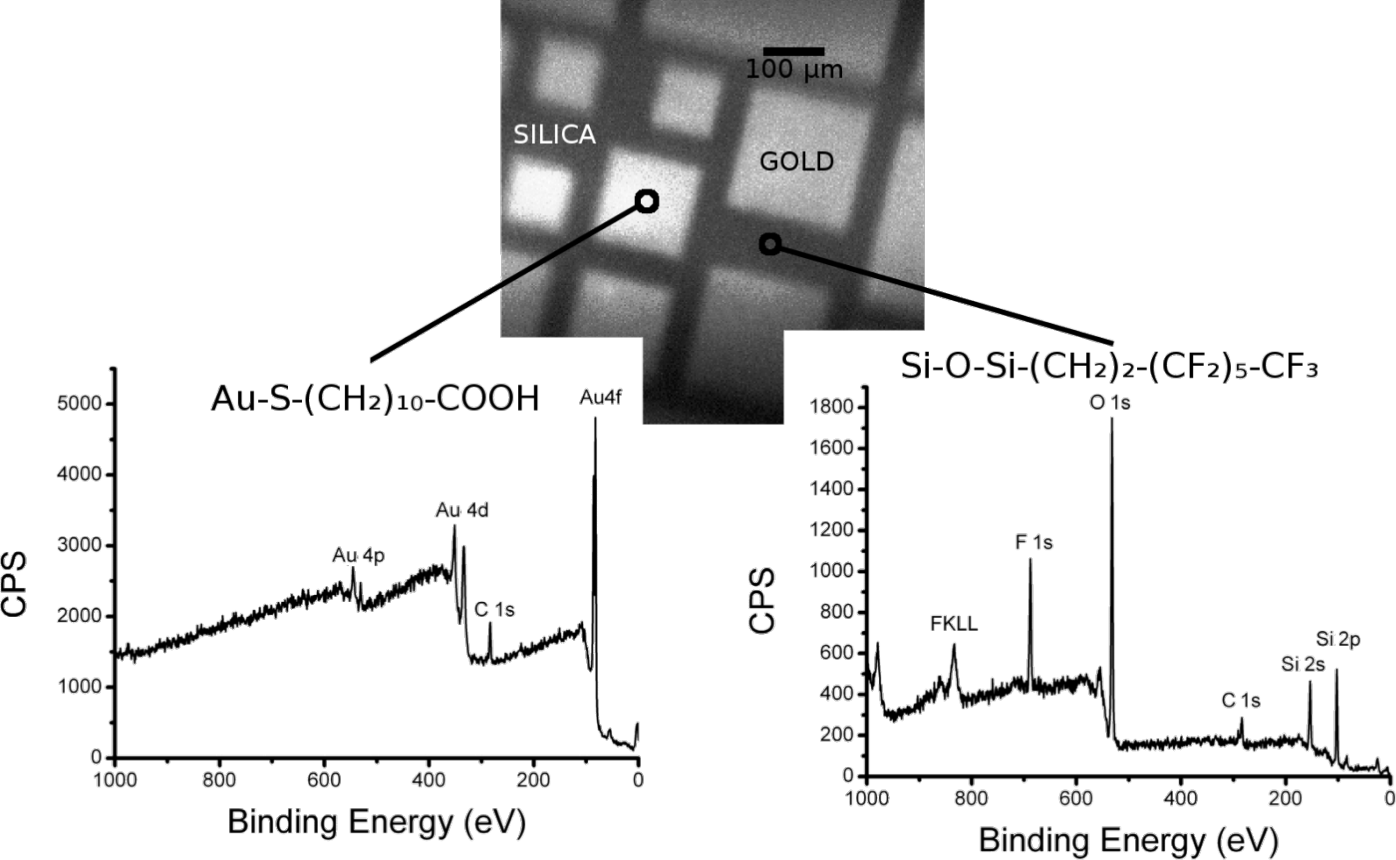
An SXI image and XPS spectra of a micropatterned gold on silica substrate sample, orthogonally functionalized with MUA and F-silane. The analyzed areas for the spectra were roughly 10 µm and their approximate localization is indicated on the image. The scale bar in the image is 100 µm.

Micropatterned gold on a silica substrate (with features of ≈100 µm) functionalized with either (1) 1*H*,1*H*,2*H*,2*H*-perfluorodecanethiol and 2-[methoxy(polyethyleneoxy)propyl]trimethoxysilane (F-thiol + PEG/Si) or (2) trichloro(1*H*,1*H*,2*H*,2*H*-perfluorooctyl)silane and 11-mercapto-1-undecanoic acid (F-silane + MUA) were analyzed using XPS. For both surfaces, an initial image was acquired using scanning X-ray imaging (SXI; X-ray beam induced secondary electron images). This allows the gold microsquares (brighter) and surrounding silica areas (darker) to be visualized, as shown in Figure 2 and Figure 3. Then, two different analysis areas (≈10 µm in diameter) corresponding to the gold and silica surfaces were selected to perform a survey spectrum. High-resolution spectra of the different peaks could not be obtained due to the low signal from the small analysis areas. These spectra show the following:

– When the sample was simultaneously functionalized with a perfluorinated thiol and a PEG/silane (Figure 2)

1. On gold (left spectrum), fluorine is clearly present as evidenced by the F 1s and F KLL peaks, showing the presence of the perfluorinated thiol (F-thiol, molecular structure given above the spectrum). Furthermore, no Si 2s or O 1s peaks were detected, verifying the absence of PEG/silane. This suggests that, on the gold areas, F-thiol is specifically grafted while PEG/Si is not adsorbed.

2. On silica (right spectrum), fluorine is clearly absent as evidenced by the lack of F 1s or F KLL peaks, verifying the absence of the F-thiol. The presence of PEG/silane cannot be assessed by the silicon or oxygen-related peaks since these are present on the silica substrate. However, the presence of the C 1s peak seems to suggest that the silane is indeed grafted, though a contribution from other sources of carbon cannot be ruled out.

– When the sample is simultaneously functionalized with F-silane and an alkylthiol (Figure 3), the orthogonality of the functionalization is proven by the same arguments as above, the main one being the presence of fluorine on silica and not on gold.

**Figure 4:**
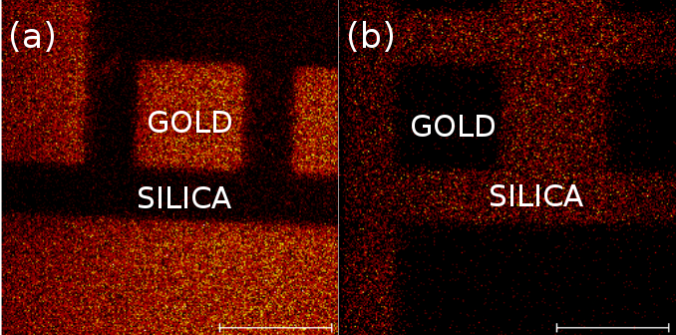
ToF–SIMS fluorine mapping of patterned gold on silica surfaces, orthogonally functionalized with F-thiol + PEG/Si (a) and F-silane + MUA (b). The scale bars are 100 μm.

We also conducted fluorine mapping on similar orthogonally functionalized surfaces using ToF–SIMS, which has been shown to be especially well-suited for the characterization of chemically patterned surfaces [29–30]. Figure 4 shows the presence of fluorine in both cases (F-thiol + PEG/Si and F-silane + MUA). In each case, only fluorine is present (or is very predominant) on the gold microsquares (Figure 4a) or the surrounding silica (Figure 4b) but not on both, which demonstrates the good orthogonality of the single-step orthogonal functionalization.

**Figure 5:**
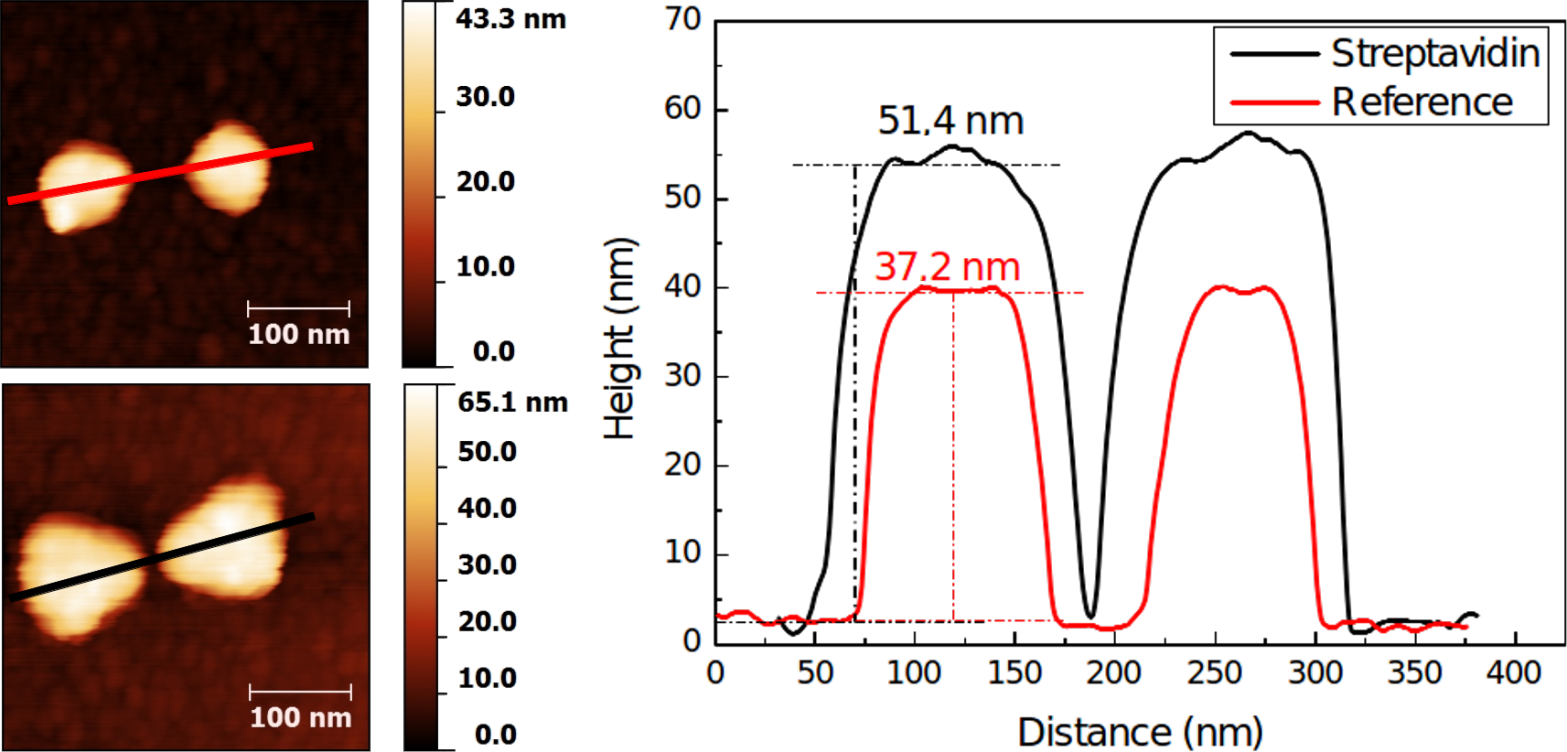
AFM height profiles of gold nanostructures on silica. The reference sample (red) was not functionalized or subjected to protein incubation and shows a height consistent with the deposition of 8 nm Ti + 30 nm Au. The streptavidin sample (black) was orthogonally functionalized and subjected to protein immobilization. The increase in size is indicative of the binding of streptavidin on the nanostructure.

Additionally, a nanostructured gold-on-silica substrate was functionalized with biotinylated thiols and antifouling PEG/silanes. A similar approach was already used to direct the immobilization of streptavidin-coated nanoparticles [31] onto the gold nanostructures. Here, “single” (i.e., not adsorbed on beads) proteins were immobilized as shown in Figure 5.

## Conclusion

The orthogonal chemical functionalization of patterned metal on dielectric surfaces appears to be a key process to direct target biomolecules onto individual nanostructures. This can be useful in different fields of nanotechnology, especially in the development of LSPR-based biosensors. In this paper, we reported different single-step functionalization procedures of patterned gold on silica surfaces with alkylthiols and silanes. The direct chemical characterization using XPS and ToF–SIMS provided evidence of the orthogonality, and AFM topography measurements showed the utility of this approach for biomolecule immobilization. Current work is being undertaken to implement this methodology into LSPR biosensors.

## Experimental

### Substrate patterning

A silica thin film (100 nm) was sputtered onto clean silicon wafers. UV lithography was used to define different patterns (lines, squares) with typical dimensions ranging from 2 to 100 µm. Electron beam lithography was used to develop the gold nanostructures (typical dimensions of 100 nm). Titanium (8 nm) and gold (30 nm) were deposited by electron beam evaporation. After lift-off, the samples were cleaned by oxygen plasma treatment (Anatech) at 400 sccm of oxygen, 350 W of forward power (10 W reflected), 90 Pa, for 5 min to ensure that no residual resist remained on the surface.

### Surface functionalization

HS-(CH_2_)_11_-NH-C(O)-Biotin 95% (MU-Biot) was purchased from ProChimia. 1*H*,1*H*,2*H*,2*H*-Perfluorodecanethiol (F-thiol) 97% was purchased from Sigma-Aldrich. Trichloro(1*H*,1*H*,2*H*,2*H*-perfluorooctyl)silane (F-silane) 97% and 2-[methoxy(polyethyleneoxy)propyl]trimethoxysilane 90% (PEG/Si, MW = 460 g/mol) were purchased from abcr. Dichloromethane (DCM) 99.9% was purchased from Sigma-Aldrich then degassed and dried over molecular sieves. The thiols and silanes were dissolved in dry DCM at room temperature in different proportions, as given in Table 1.

**Table 1:** Thiol/silane mixtures used for orthogonal functionalization in 25 mL of DCM.

Compound	Quantity	Molar concentration (approx.)

MUA + F-silane	50 mg/10 µL	9 mM/1 mM
F-thiol + PEG/Si	100 µL/10 µL	14 mM/1 mM
MU-Biot + PEG/Si	50 mg/10 µL	5 mM/1 mM

After plasma cleaning, the resulting gold oxide is unstable and the samples were allowed to deoxidize for 24 h in fluoroware. Then, the samples were immersed in thiol/silane solutions under nitrogen and allowed to react for 48 h. The samples were then rinsed two times with fresh DCM for 5 min under sonication (Branson, 42 kHz, 100 W) followed by a stream of ultrapure water and dried with nitrogen.

### Characterization

#### XPS

XPS characterization was conducted using an ULVAC-PHI VersaProbe II spectrometer equipped with a monochromatic Al Kα X-ray source (1486.6 eV). The analysis area can be adjusted from 200 µm to 10 µm and the energy scale was calibrated with reference to the C 1s line at a binding energy of 284.8 ± 0.1 eV (C–C/C–H). The charging effect is controlled by a dedicated neutralizer using a combination of ions and electrons at very low energy (0.1 eV). The X-ray spot can be scanned with a field of view of 1300 µm. This instrument allows for the recording of both XPS spectra and SXI images.

#### ToF–SIMS

ToF–SIMS measurements were performed with a Physical Electronics (Chanhassen, USA), TRIFT III instrument operated with a pulsed 22 keV Au ion gun (ion current of 2 nA). Areas of 300 × 300 µm were scanned. Under the present operation conditions, the lateral resolution is on the order of 1 μm. Submicron resolution can be achieved, albeit hindering mass resolution. The ion dose was kept below the static conditions limits. The data were analyzed using WinCadence software. The mass calibration was performed on hydrocarbon secondary ions.
